# A Pilot Study on Whether Concept Mapping as a Teaching Tool Improves Student Engagement in Phase I Bachelor of Medicine and Bachelor of Surgery (MBBS) Biochemistry Lecture Sessions

**DOI:** 10.7759/cureus.114378

**Published:** 2026-08-11

**Authors:** Jyotchna D Bade, L. Reshma Shireesha, BVK Vijayalakshmi, Nagarjuna Sivaraj, Sitanshu Sekhar Kar, Zayapragassarazan Z, Venkataramana Kandi

**Affiliations:** 1 Biochemistry, Great Eastern Medical School and Hospital, Srikakulam, IND; 2 Physiology, Government Medical College, Srikakulam, Srikakulam, IND; 3 Physiology, Government Medical College, Paderu, Paderu, IND; 4 Research and Development, Great Eastern Medical School and Hospital, Srikakulam, IND; 5 Preventive and Social Medicine, Jawaharlal Institute of Postgraduate Medical Education and Research, Puducherry, IND; 6 Medical Education, Jawaharlal Institute of Postgraduate Medical Education and Research, Puducherry, IND; 7 Clinical Microbiology, Prathima Institute of Medical Sciences, Karimnagar, IND

**Keywords:** active learning, biochemistry pedagogy, biochemistry teaching, competency-based medical education (cbme), concept mapping, medical education, undergraduate mbbs

## Abstract

Introduction

Engaging Phase I Bachelor of Medicine and Bachelor of Surgery (MBBS) students in biochemistry lectures is often challenging due to the abstract nature of metabolic concepts. Concept mapping, an active learning strategy that visualizes hierarchical relationships, has been proposed to enhance meaningful learning and critical thinking. This pilot study evaluated the effectiveness of concept mapping in improving student engagement and academic performance compared to traditional didactic instruction.

Methods

An exploratory mixed‑methods pilot study was conducted among Phase I MBBS students (N=98) randomly allocated into two equal groups: intervention group (n=49), taught via concept mapping, and control group (n=49), taught via traditional lectures. Pre‑ and post‑intervention knowledge scores, internal assessment marks, and student perception surveys were analyzed. Statistical methods included independent samples t-tests, paired t-tests, 2×2 repeated‑measures analysis of variance (RM‑ANOVA), and thematic analysis of qualitative feedback. A significance threshold of p<0.05 was applied.

Results

At baseline, pre‑test knowledge scores were comparable between groups (24.22±6.78 vs. 23.85±6.42; p=0.780), confirming homogeneity. Post‑intervention, the concept mapping group achieved significantly higher mean scores (65.24±10.72 vs. 42.10±8.15; p<0.001) and a markedly greater absolute knowledge gain (Δ=+41.02±8.20 vs. +18.25±5.10; p<0.001). RM‑ANOVA revealed a highly significant group×time interaction effect (F(1,96)=272.58; p<0.001; partial η^2^=0.74), confirming that improvements were specifically attributable to concept mapping rather than routine curricular progression. The intervention group also outperformed controls in formative internal assessments (p<0.001). Qualitative feedback indicated that over 80% of students perceived concept mapping as enhancing engagement, conceptual clarity, and exam preparation.

Conclusion

This pilot study provides initial feasibility evidence that concept mapping is an engaging, practical pedagogical strategy in Phase I MBBS biochemistry, associated with measurable short-term gains in knowledge acquisition and internal assessment performance compared to traditional lectures. While these findings support integrating structured mapping activities into competency-based medical education (CBME) frameworks, future multicenter studies with extended follow-up are needed to confirm long-term retention and clinical translational benefits.

## Introduction

Biochemistry serves as a critical foundation in undergraduate medical education by providing Phase I Bachelor of Medicine and Bachelor of Surgery (MBBS) students with essential knowledge regarding the molecular basis of health and disease. However, delivering biochemistry instruction effectively remains challenging due to an extensive syllabus, abstract principles, complex metabolic pathways, and the need to bridge basic science with clinical application [1].

Traditional didactic lectures have historically dominated biochemistry instruction. While efficient for delivering volume, passive lecture environments often fail to foster active engagement, leading to poor knowledge retention and difficulties in correlating biochemical concepts [2]. In response, modern competency-based medical education (CBME) frameworks emphasize student-centered active learning strategies to foster analytical reasoning, long-term retention, and collaborative learning.

Concept mapping, originally conceptualized by Joseph D. Novak, is a graphical strategy designed to organize and represent knowledge [3,4]. Utilizing nodes (representing core concepts) and links (defining relationships), concept maps depict hierarchical structures that align with Ausubel's Theory of Meaningful Learning, which asserts that learning occurs when new information is explicitly integrated with existing cognitive frameworks [3,4].

Recognition of concept mapping as an educational technique in health professions has grown due to its association with active student engagement and critical thinking [5]. By visual mapping of metabolic pathways, students can categorize concepts to connect enzymes, disease processes, and regulatory mechanisms [6]. However, limited empirical evidence exists evaluating the utility and success of incorporating concept mapping directly into Phase I MBBS biochemistry lectures [7-9]. Therefore, this pilot study was undertaken to evaluate the effectiveness of concept mapping in improving student engagement and academic performance, as well as to assess student perceptions of this instructional method.

## Materials and methods

Study design and setting

This exploratory pilot study utilized a mixed-methods approach to assess the utility of concept mapping. The study was conducted over a six-month duration during the 2023-2024 academic year in the Department of Biochemistry at Great Eastern Medical School and Hospital in Srikakulam, India. Ethical approval was obtained from the institute's Institutional Ethics Committee (approval number: 160/IEC/GEMS&H/2023) prior to study initiation, and all participants provided written informed consent. 

Study population and eligibility criteria

The study population comprised Phase I MBBS undergraduate medical students enrolled in the Department of Biochemistry at Great Eastern Medical School and Hospital during the 2023-2024 academic year who provided voluntary written informed consent and completed both the pre- and post-test evaluations. Students were excluded from the study if they demonstrated prior formal exposure to or practical proficiency in constructing concept maps on the baseline pre-test questionnaire, were absent during any of the educational intervention sessions (concept mapping or traditional lectures), missed any scheduled formative or internal academic assessments during the six-month study period, or declined/withdrew consent to participate.

Participant selection and randomization

A total of 98 Phase I MBBS students voluntarily agreed to participate. Students missing either the educational intervention or assessment sessions were excluded. Participants were randomly allocated in a 1:1 ratio into two parallel groups: intervention group (n=49; received instruction using concept mapping techniques) and control group (n=49; received instruction via traditional didactic lectures).

Sample size justification

In accordance with the Consolidated Standards of Reporting Trials (CONSORT) 2010 extension for pilot and feasibility trials, a formal a priori power calculation to estimate sample size was deliberately omitted. Pilot studies primarily aim to evaluate pedagogical feasibility, assess methodology, and gather preliminary effect size estimates rather than test definitive hypotheses. The sample size (n=98; n=49 per group) was determined based on a convenience sample of all eligible, consenting Phase I MBBS students enrolled in the department during the study period. This sample size exceeds the minimum recommendation of 12-30 participants per group generally cited for pilot trials to reliably estimate parameters (e.g., standard deviation and effect size) required for future definitive power calculations.

Educational intervention

Before implementation, participating faculty members underwent training to construct and standardize concept map delivery. Topics selected for the intervention included complex and interrelated areas of biochemistry: protein structure and amino acid classification/reactions.

Intervention Group

Students received introductory orientation on constructing concept maps (using carbohydrate classification as an exemplar) incorporating nodes, directional arrows, and linking phrases. Subsequently, sessions involved active map construction and interactive student-led discussions explaining relationships between metabolic pathways.

Control Group

Students received equivalent syllabus coverage through traditional lecture presentation slides and standard blackboard instruction without mapping exercises.

Implementation Protocols and Timing

The educational intervention was delivered across six structured bi‑weekly sessions, totaling six lecture hours, over the six‑month study period. Each session was 60 minutes in duration and followed a standardized time‑allocation protocol to ensure consistency across groups. During the first 15 minutes, the instructor provided an interactive presentation of core theoretical concepts using standardized slides. This segment was designed to establish foundational knowledge and orient students to the session's learning objectives. The subsequent 30 minutes differed by group allocation. In the intervention group, students worked in small teams of 4-5 to actively construct concept maps. These maps incorporated nodes, directional arrows, and linking phrases and were facilitated by trained faculty using standardized guidance prompts. In contrast, the control group received a traditional didactic lecture extension, supplemented by blackboard diagrams and individual note‑taking, covering identical learning objectives. The final 15 minutes of each session were dedicated to formative review and student participation. Intervention group students presented selected concept maps, while control group students summarized content through instructor‑led slides. This closing segment reinforced learning outcomes and provided opportunities for clarification through questions and answers.

Assessor Blinding and Standardization

To minimize observer and evaluation bias, all routine formative and internal examination scripts were anonymized with unique numerical identification codes. Outcome assessors (faculty grading the case-based questions, short-answer questions, and pre-/post-tests) were completely blinded to participant group allocation. Standardized answer keys and defined scoring criteria were utilized across all assessments.

Assessment instruments

Data collection was conducted using two structured assessment instruments (see Appendix A and Appendix B).

Pre-test Assessment

This instrument was administered prior to the intervention to establish baseline familiarity with concept mapping concepts (questions 1-2 and 9) and measure baseline conceptual knowledge using a six-item multiple-choice and true/false tool (questions 3-8) (see Appendix A).

Post-test Assessment 

Administered following the six-month intervention period, this instrument evaluated both post-intervention knowledge acquisition and student engagement (see Appendix B). Core knowledge gain was evaluated using the identical six-item knowledge set from the pre-test (questions 3-8) to allow direct pre- vs. post-test comparisons. Student engagement, enjoyment, and practical application during internal exam preparation were evaluated using distinct Perception and Engagement items (questions 1, 2, and 9) (Appendix B).

Instruments validation

The study instruments (Appendices A, B, and C) underwent comprehensive evaluation for content validity, face validity, and reliability to ensure pedagogical rigor. Content validity was quantitatively established by an expert panel of six faculty moderators from the Jawaharlal Institute of Postgraduate Medical Education and Research (JIPMER), who reviewed, refined, and validated the tools prior to administration. Using a 4‑point ordinal scale (1=not relevant; 4=highly relevant), Item‑Content Validity Index (I‑CVI) values ranged from 0.83 to 1.00, while Scale‑Level CVI (S‑CVI) values were 0.94 for the Pre‑Intervention (Appendix A) and Post‑Intervention (Appendix B) instruments and 0.90 for the structured feedback questionnaire (Appendix C). All values exceeded the recommended threshold of 0.80, confirming strong content validity, pedagogical clarity, and domain alignment across survey items. Face validity was further established through pilot testing with a representative group of Phase I MBBS students (n=10; excluded from the final cohort), ensuring the clarity and readability of items. Reliability analysis demonstrated high internal consistency for perception items, with a Cronbach's alpha (α) of 0.84. Test‑retest reliability was intentionally omitted to avoid recall bias and testing artifacts, given the active intervention design.

Qualitative Analysis Procedure

Qualitative responses from the open‑ended feedback items (Appendix C) were analyzed using Braun and Clarke's six‑phase thematic analysis framework. Two independent qualitative researchers systematically and iteratively reviewed the raw transcripts to generate initial codes, collate them into broader prospective themes, and refine theme definitions. Any discrepancies in coding or theme assignment were resolved through consensus discussions with a third senior researcher, ensuring methodological rigor. Inter‑coder reliability was established with a calculated Cohen's kappa coefficient (κ=0.86), reflecting a high level of coding agreement and consistency across raters.

Outcome measures and data collection

Baseline and Knowledge Gain

Baseline knowledge and familiarity with concept mapping components were assessed before (pre-test) and after (post-test) the educational module.

Academic Performance

Performance was evaluated through routinely scheduled formative and internal assessments comprising case-based questions, multiple-choice questions (MCQs), and short-answer questions covering the targeted biochemistry topics.

Student Perception

At the conclusion of the module, participants in the intervention group completed a validated feedback questionnaire consisting of Likert-scale items (measuring attentiveness, interest, and understanding) and open-ended items (capturing subjective pros, cons, and suggestions) (see Appendix C).

Statistical analysis

All raw data, including pre‑ and post‑test scores, internal assessment marks, and student perception survey responses, were initially entered and organized using Microsoft Excel (Microsoft Corporation, Redmond, Washington, United States). Data cleaning and descriptive statistics (mean, standard deviation, frequencies, and percentages) were performed in Excel, while inferential analyses were conducted using IBM SPSS Statistics for Windows, Version 25.0 (IBM Corp., Armonk, New York, United States). Paired t‑tests were applied to compare pre‑ and post‑test scores within the intervention group, and independent samples t‑tests were used to compare internal assessment scores between the intervention and control groups. Baseline comparability between groups was evaluated using an independent samples t‑test on pre‑test knowledge scores prior to the educational intervention. To further examine the interaction effect between instructional method (intervention vs. control) and time (pre‑test vs. post‑test), a 2×2 repeated‑measures analysis of variance (RM‑ANOVA) was performed. In addition, absolute knowledge gain (post‑test score minus pre‑test score) was calculated for each participant and compared between groups using an independent samples t‑test. A p‑value of <0.05 was considered statistically significant. Survey data were summarized descriptively without inferential testing.

## Results

At baseline, both groups demonstrated comparable pre‑intervention knowledge scores, with no statistically significant difference between them (intervention group: 24.22±6.78; control group: 23.85±6.42; t(96)=0.28; p=0.780), thereby confirming homogeneity across study arms prior to the intervention. Over the six‑month study period, both groups showed improvement; however, the absolute knowledge gain (Δ) was significantly greater in the concept mapping group (+41.02±8.20) compared to the control group (+18.25±5.10; t(96)=-16.51; p<0.001). Within the intervention group (n=49), the mean pre‑test score of 24.22±6.78 increased substantially to 65.24±10.72 in the post‑test, with the difference reaching high statistical significance (p<0.001). Furthermore, the 2×2 RM-ANOVA revealed a statistically significant group×time interaction effect (F(1,96)=272.58; p<0.001; partial η^2^=0.74), confirming that the observed knowledge improvement was specifically attributable to the concept mapping methodology rather than routine teaching or maturation over time (Table 1).

**Table 1:** Comparison of pre-test, post-test, and absolute knowledge gain scores between the control and intervention groups (N=98) p-value: probability value; SD: standard deviation; df: degrees of freedom; t-value: Student's t-test value; RM-ANOVA: repeated‑measures analysis of variance Intergroup baseline and post‑intervention scores were compared using two‑tailed independent samples t-tests (df=96). Absolute knowledge gain (Δ) was calculated as the difference between post‑test and pre‑test scores. Overall intervention effectiveness across time points was further evaluated using a 2×2 RM-ANOVA, which revealed a highly significant group×time interaction effect (F(1,96)=272.58; p<0.001; partial η^2^=0.74). A p-value of <0.05 was considered statistically significant.

Parameter/assessment phase	Control group (n=49) mean±SD	Concept mapping group (n=49) mean±SD	Between-group difference (t-value)	df	P-value
Pre-test (baseline)	23.85±6.42	24.22±6.78	-0.28	96	0.780
Post-test (6 months)	42.10±8.15	65.24±10.72	-11.98	96	<0.001
Absolute knowledge gain (Δ)	+18.25±5.10	+41.02±8.20	-16.51	96	<0.001

Comparison of internal assessment scores revealed that the concept mapping group achieved higher performance (46.15±4.90) than the traditional lecture group (42.75±4.60). This intergroup difference was statistically significant (p<0.001), indicating a sustained benefit of concept mapping (Table 2).

**Table 2:** Comparison of internal and formative assessment scores between the control and intervention groups (N=98) p-value: probability value; SD: standard deviation; t-value: Student's-t test value; df: degrees of freedom Intergroup comparisons were analyzed using independent samples t‑tests. A p-value of <0.05 was considered statistically significant.

Assessment	Control group (n=49) mean±SD	Concept mapping group (n=49) mean±SD	t-value	df	P-value
1st internal assessment	42.75±4.60	46.15±4.90	-3.54	96	<0.001
2nd internal assessment	39.14±4.80	42.48±5.00	-3.37	96	<0.001
Final internal assessment	43.78±5.10	47.56±5.20	-3.63	96	<0.001

Feedback survey analysis showed that a majority of students reported positive perceptions of concept mapping. Over 80% agreed that it improved clarity of biochemistry concepts, enhanced engagement, and facilitated better retention, reflecting strong acceptance of the method (Table 3).

**Table 3:** Student perception regarding concept mapping (n=49) Responses to the perception survey were summarized as frequencies and percentages. Descriptive statistics were used to present categorical data. No inferential statistical tests were applied, as the survey was designed to capture student feedback and engagement trends.

Feedback statement	Strongly agree/agree n (%)	Neutral (%)	Disagree/strongly disagree (%)
Concept mapping improved interest and engagement in biochemistry lectures	40 (81.60)	2 (4.10)	7 (14.30)
Concept mapping helped in understanding complex biochemical concepts	44 (89.80)	2 (4.10)	3 (6.10)
Concept mapping should be continued in biochemistry teaching	46 (94)	0 (00)	3 (6.10)
Concept mapping was useful for preparation for internal examinations	46 (94)	1 (2.10)	2 (4.10)

In summary, the quantitative and survey data demonstrate that concept mapping improved both academic performance and student engagement. These objective gains are further illustrated by representative student-drawn concept maps (Appendix D) and substantiated by qualitative feedback detailing the student experience.

A qualitative evaluation of the open-ended responses provided by participants in the intervention group (n=49) revealed overarching themes categorized into perceived benefits (pros), observed challenges (cons), and student recommendations (suggestions). Thematic analysis showed that students predominantly valued concept mapping for enhancing visual clarity, encouraging active classroom involvement, and serving as a concise tool for exam revision. Conversely, minor challenges were reported regarding the initial time investment required to draw maps and the difficulty of representing highly non-linear or detailed pathways. Students recommended expanding the strategy across more topics and providing instructor-verified reference maps prior to examinations. Table 4 summarizes the thematic categorization of qualitative student feedback along with representative participant quotes.

**Table 4:** Summary of the thematic analysis of qualitative feedback from the intervention group (n=49) MBBS: Bachelor of Medicine and Bachelor of Surgery

Primary theme	Sub-theme/category	Representative student quotes	Actionable suggestions/insights
Perceived benefits (pros)	Active engagement and retention	Concept mapping kept me active during the lecture instead of just listening passively. Drawing the pathways helped me retain complex metabolic links better	Fosters student-centered active learning and higher classroom attentiveness
	Conceptual clarity	Connecting enzymes, substrates, and clinical conditions visually made carbohydrate pathways much easier to understand	Replaces rote memorization with structural mental organization
	Efficient exam revision	It acts as a quick revision summary right before tests rather than going through pages of textbook notes	Serves as an effective high-yield study aid during internal assessments
Observed challenges (cons)	Content complexity limitations	Some biochemistry topics are too dense or detailed to fit neatly into a single concept map	Complex multi-step pathways may require sub-mapping or structured hybrid diagrams
	Initial effort and time constraints	Designing a proper map takes more time initially until you get used to connecting nodes and linking words	Requires introductory orientation and guided practice sessions to reduce cognitive load
Student suggestions	Curriculum expansion	Concept mapping should be extended to all major biochemistry topics like protein metabolism and clinical correlations	Integrate concept mapping systematically into routine Phase I MBBS lectures
	Instructor guidance	It would be very helpful if teachers could provide standard, teacher-verified concept maps at the end of every topic for reference	Provide expert-reviewed master maps alongside student-generated maps for final exam preparation

## Discussion

This pilot study evaluated the impact of concept mapping as an active learning strategy to enhance engagement and academic performance among Phase I MBBS students during biochemistry lectures. At baseline, both cohorts demonstrated comparable pre‑intervention knowledge scores (p=0.780), confirming homogeneity across study arms. Following implementation, students in the concept mapping group achieved significantly greater post‑intervention scores (65.24±10.72 vs. 42.10±8.15; p<0.001) and a markedly higher absolute knowledge gain (+41.02±8.20 vs. +18.25±5.10; p<0.001). The 2×2 RM-ANOVA revealed a highly significant group×time interaction effect (F(1,96)=272.58; p<0.001; partial η^2^=0.74), confirming that the observed improvements were specifically attributable to the structured concept mapping methodology rather than routine curricular progression or maturation over time. The dramatic increase in scores from pre‑test (24.22±6.78) to post‑test (65.24±10.72; p<0.001) underscores the effectiveness of concept mapping in fostering meaningful visual learning, structural knowledge retention, and enhanced cognitive processing. While these quantitative gains are encouraging, they represent preliminary, single-center pilot evidence and must be interpreted within the context of study design constraints, specifically, that participant blinding was unfeasible given the active nature of the intervention, alongside potential testing effects [10-12].

Comparable improvements have been reported in prior studies. Farrand et al. observed that medical students using mind maps achieved a 12% higher recall rate compared to those using traditional note-taking [13]. Novak and Cañas emphasized that hierarchical knowledge organization connects new information with existing cognitive structures, thereby promoting meaningful learning [10]. Several studies demonstrated that concept maps improved critical reasoning, with students scoring 15-20% higher in integrating basic science into clinical problem-solving compared to controls [12,14,15]. The statistically significant advantage observed in formative and internal assessments (p<0.05) in our study supports the premise that visual organizational tools enhance higher-order cognitive processing, aligning with Freeman et al., who reported that active learning strategies increased exam performance by 6% across 225 STEM courses [16].

Traditional didactic lectures often encourage rote memorization without conceptual integration, leading to passive learning environments. In contrast, concept mapping shifts the paradigm toward active knowledge construction, where learners identify relationships, categorize material, and synthesize biochemical pathways into coherent frameworks. This transition is consistent with CBME principles, which emphasize analytical thinking, problem-solving, and clinical application of knowledge [17].

Survey data reinforced these quantitative outcomes. More than 80% of participants in our study reported increased attentiveness, engagement, and improved ability to grasp challenging biochemical concepts [18]. Kassab and Hussain similarly found that 87% of students in a problem-based curriculum perceived concept mapping as enhancing their understanding [18]. Qualitative feedback corroborated these findings, with students noting that visual mapping reduced confusion, clarified complex pathways, and fostered self-directed learning [19]. Abel and Freeze reported parallel outcomes in nursing education, where 92% of students agreed that concept mapping improved their ability to organize and retain knowledge [19].

Importantly, learner-centered approaches such as concept mapping resonate with National Medical Commission (NMC) directives, which advocate collaborative learning, communication skills, and integration of basic sciences with clinical reasoning [20]. Spencer and Jordan emphasized that learner-centered strategies improved communication and teamwork, with over 70% of students reporting enhanced collaborative skills [20]. Leon et al. underscored the importance of pilot studies in guiding larger-scale clinical and educational research, noting that pilot data often predicts feasibility and informs sample size calculations for definitive trials [21]. Tandon et al. further highlighted that concept maps improved analytical thinking, with undergraduate medical students demonstrating a 20% increase in problem-solving scores after structured mapping sessions [22].

Collectively, these exploratory pilot findings suggest that concept mapping is a promising pedagogical strategy for undergraduate medical education. While further multicenter research is required to evaluate long-term retention and clinical translation, this study provides valuable feasibility data to guide future definitive trials.

Study limitations

This study has several limitations related to scope, internal validity, and methodology. First, as an exploratory pilot study conducted at a single institution, a formal a priori sample size calculation was not performed. Instead, a pragmatically derived sample (n=49 per group) was used to assess pedagogical feasibility, which restricts generalizability and statistical power. Second, although outcome assessors were blinded during grading, participant blinding was inherently impossible due to the active nature of the educational strategy. This limitation introduces the possibility of performance or novelty bias among students. Third, repeated administration of identical pre‑ and post‑test instruments may have contributed to a testing effect or memory recall bias. However, the six‑month gap between evaluations likely mitigated immediate recall. Fourth, despite standardizing session durations, differences in time‑on‑task and active cognitive effort between interactive concept mapping and traditional lecturing could represent an unmeasured confounding variable. Fifth, the intervention was restricted to selected biochemistry topics delivered over six months, limiting the ability to evaluate long‑term retention and clinical translational skills. Finally, although qualitative feedback was analyzed using Braun and Clarke's framework, reliance on self‑reported surveys introduces the potential for response bias. Future research should therefore involve multicenter randomized controlled trials with extended follow‑up, standardized time‑on‑task controls, and alternate evaluation instruments to address these validity threats and strengthen external generalizability.

## Conclusions

This pilot study provides initial feasibility evidence supporting concept mapping as an engaging pedagogical strategy in Phase I MBBS biochemistry, associated with measurable short-term improvements in knowledge acquisition and internal assessment performance compared to traditional didactic lectures. These findings provide strong empirical evidence supporting a transition from passive lecturing toward active, student-centered learning methodologies in undergraduate medical education, offering medical educators and curriculum decision-makers a clear rationale to integrate structured mapping activities into core biochemistry modules in alignment with NMC CBME directives. Looking forward, these results should encourage institutional adoption through faculty training in standardized concept map facilitation, the development of verified "master maps" for exam revision, and multicenter longitudinal studies with larger cohorts to evaluate long-term knowledge retention and its translation into clinical reasoning during advanced phases of medical training.

## Figures and Tables

**Figure 1:**
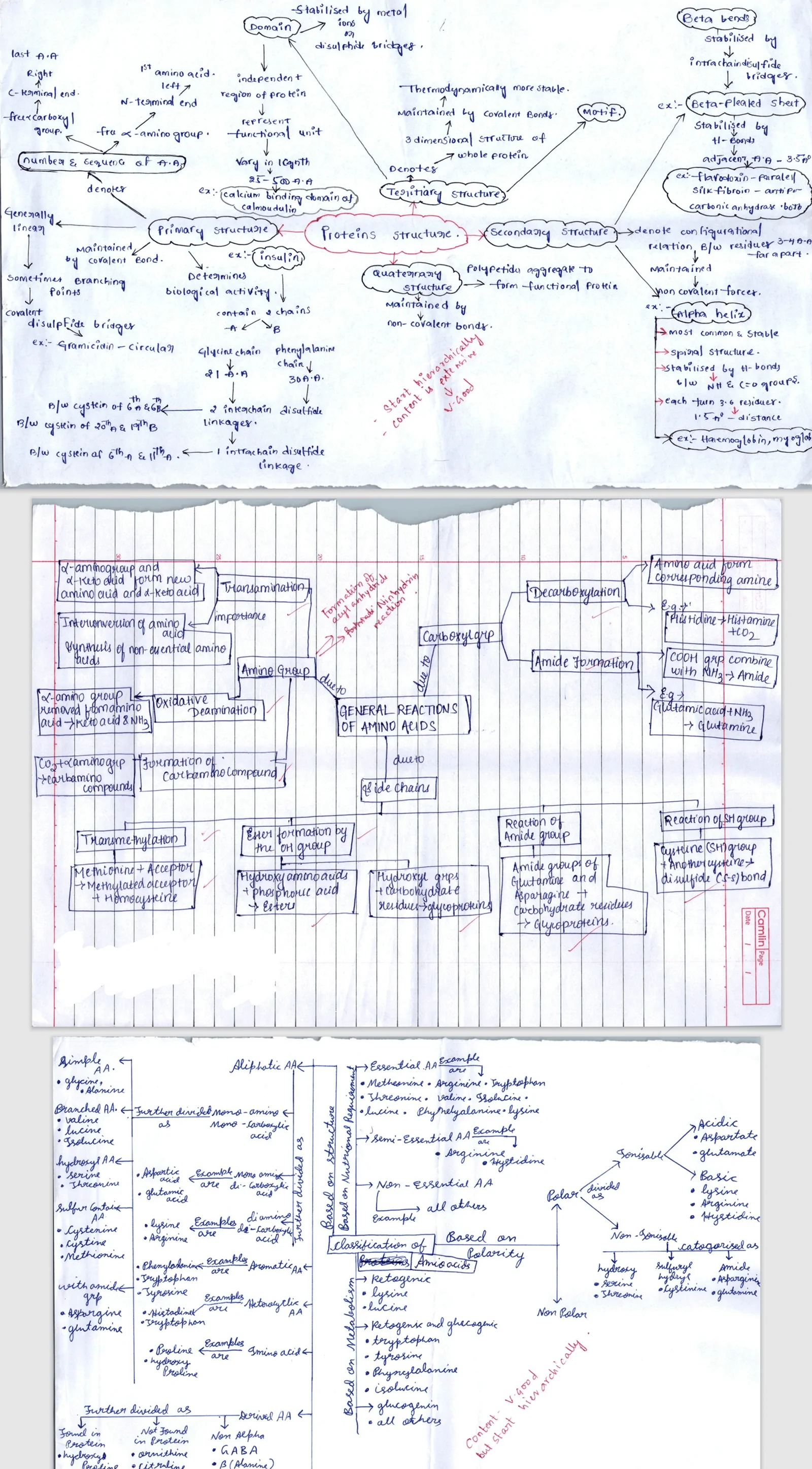
Concept maps self-prepared by the students

## Appendices

Appendix A

Pre-intervention Assessment Tool

**Table 5:** Pre-intervention assessment tool

Question number	Question item	Response format/options	Target concept/objective
1	Have you ever heard of a concept map?	Yes	Baseline awareness
		No	
2	If yes, define it in your own words.	Free-text open response	Baseline conceptual understanding
3	Concept mapping is primarily a passive learning technique that doesn't engage students actively.	Yes	Passive vs. active learning perception
		No	
4	Which steps typically come first when creating a concept map?	(a) Add connecting lines	Step-by-step procedural knowledge
		(b) Defining key concepts	
		(c) Color code the map	
		(d) Choose paper size	
5	Which of the following is NOT a common use of concept mapping?	(a) Summarizing information	Understanding application scope
		(b) Brainstorming ideas	
		(c) Memorizing facts	
		(d) Planning a research project	
6	In a concept map, concepts are usually represented as circles or ovals, and connecting lines indicate relationships.	Yes	Structural component awareness
		No	
7	In a concept map, what do connecting lines with arrows indicate?	(a) No relationship	Understanding relational links
		(b) Hierarchical relationship	
		(c) Causative relationship	
		(d) Opposite relationship	
8	Concept maps are a one-time tool, and once created, they cannot be modified or revised.	True	Understanding iterative nature
		False	
9	When creating a concept map, what type of word or phrase is typically used to label connecting lines?	(a) Specific numbers	Procedural knowledge of linking phrases
		(b) Arrows	
		(c) Linking phrases/prepositions	
		(d) Symbols	

Appendix B

Post-intervention and Student Engagement Evaluation Tool

**Table 6:** Post-intervention assessment, engagement, and practical application tool

Section	Question number	Question item	Response options
Section I: Engagement and perception	1	Did you enjoy using concept mapping during lectures?	Yes
			No
	2	How likely are you to apply concept mapping for further studying?	(a) Very likely
			(b) Likely
			(c) Neutral
			(d) Unlikely
			(e) Very unlikely
Section II: Knowledge assessment	3	Concept mapping is primarily a passive learning technique that doesn't engage students actively.	Yes
			No
	4	Which steps typically come first when creating a concept map?	(a) Add connecting lines
			(b) Defining key concepts
			(c) Color code the map
			(d) Choose paper size
	5	Which of the following is NOT a common use of concept mapping?	(a) Summarizing information
			(b) Brainstorming ideas
			(c) Memorizing facts
			(d) Planning a research project
	6	In a concept map, concepts are usually represented as circles or ovals, and connecting lines indicate relationships.	Yes
			No
	7	In a concept map, what do connecting lines with arrows indicate?	(a) No relationship
			(b) Hierarchical relationship
			(c) Causative relationship
			(d) Opposite relationship
	8	Concept maps are a one-time tool, and once created, they cannot be modified or revised.	True
			False
Section III: Exam preparation and application	9	Did you use concept mapping while preparing for your first internal assessment?	Yes
			No

Appendix C

Feedback Questionnaire

**Table 7:** Structured feedback survey items

Domain	Survey item/question	Response scale/type
Importance of concept mapping	On a scale of 1-5, how would you rate the importance of concept mapping in understanding medical concepts?	1 (not important) to 5 (extremely important)
Usefulness of the study	Did the study enhance your understanding of the subject matter?	Very much
		Somewhat
		Neutral
		Not much
		Not at all
	Share specific examples of how concept mapping sessions have been useful to you.	Open-ended text
Presenter's knowledge and style	On a scale of 1-5, how would you rate the presenter's knowledge of the subject matter?	1 (poor) to 5 (excellent)
	How would you describe the presenter's style (e.g., engaging, clear, interactive)?	Open-ended text
	Was the concept presented in a manner that was easy to follow?	Yes
		No
		Comments
Constructive feedback	Were there specific topics or concepts you wished were covered in more detail?	Open-ended text
	Are there any additional resources or materials you wish the presenter had provided?	Open-ended text
Overall satisfaction	On a scale of 1-5, how satisfied are you with the overall quality of the concept mapping session?	1 (very dissatisfied) to 5 (very satisfied)
Suggestions and comments	Are there any specific suggestions you have for improving future concept mapping sessions?	Open-ended text
	Additional comments or thoughts about the session.	Open-ended text

Appendix D

Concept Maps Prepared by the Students

The representative concept maps were prepared by Phase I MBBS students during the biochemistry lecture sessions. These visual artifacts illustrate how concept mapping facilitated active engagement and improved the clarity of complex biochemical concepts (Figure 1).

## References

[1] Daley BJ, Torre DM (2010). Concept maps in medical education: an analytical literature review. Med Educ.

[2] Sannathimmappa MB, Nambiar V, Aravindakshan R (2022). Concept maps in immunology: a metacognitive tool to promote collaborative and meaningful learning among undergraduate medical students. J Adv Med Educ Prof.

[3] Hung CH, Lin CY (2015). Using concept mapping to evaluate knowledge structure in problem-based learning. BMC Med Educ.

[4] Baliga SS, Walvekar PR, Mahantshetti GJ (2021). Concept map as a teaching and learning tool for medical students. J Educ Health Promot.

[5] West DC, Pomeroy JR, Park JK, Gerstenberger EA, Sandoval J (2000). Critical thinking in graduate medical education: a role for concept mapping assessment?. JAMA.

[6] Pintoi AJ, Zeitz HJ (1997). Concept mapping: a strategy for promoting meaningful learning in medical education. Med Teach.

[7] Vink SC, Van Tartwijk J, Bolk J, Verloop N (2015). Integration of clinical and basic sciences in concept maps: a mixed-method study on teacher learning. BMC Med Educ.

[8] D'Antoni AV, Zipp GP, Olson VG, Cahill TF (2010). Does the mind map learning strategy facilitate information retrieval and critical thinking in medical students?. BMC Med Educ.

[9] Sajadi AS, Babajani A, Maroufi SS, Sarraf N (2024). Using the mind map method in medical education, its advantages and challenges: a systematic review. J Educ Health Promot.

[10] Novak JD, Cañas AJ (2026). The theory underlying concept maps and how to construct them. Florida Institute for Human and Machine Cognition, Pensacola, FL.

[11] Alhomaidan AM (2015). The effectiveness of concept mapping on learning: a study in a Saudi college-level context. Am J Educ Res.

[12] Surapaneni KM, Tekian A (2013). Concept mapping enhances learning of biochemistry. Med Educ Online.

[13] Farrand P, Hussain F, Hennessy E (2002). The efficacy of the 'mind map' study technique. Med Educ.

[14] Markow RJ, Lonning RA (1998). Usefulness of concept maps in college chemistry laboratories: students' perceptions and effects on achievement. J Res Sci Teach.

[15] Chikkarahalli Srikantaiah V, Mathada Vamadevaiah R, Gowdappa Doddawad V (2025). Enhancing anatomy learning: a concept map-based approach for first-term MBBS students. Educ Med.

[16] Freeman S, Eddy SL, McDonough M, Smith MK, Okoroafor N, Jordt H, Wenderoth MP (2014). Active learning increases student performance in science, engineering, and mathematics. Proc Natl Acad Sci U S A.

[17] Kurien M, Thomas K (2024). Competency Based Medical Education (CBME) in India: clinicians' multifaceted challenges. Indian J Otolaryngol Head Neck Surg.

[18] Kassab SE, Hussain S (2010). Concept mapping assessment in a problem-based medical curriculum. Med Teach.

[19] Abel WM, Freeze M (2006). Evaluation of concept mapping in an associate degree nursing program. J Nurs Educ.

[20] Spencer JA, Jordan RK (1999). Learner centred approaches in medical education. BMJ.

[21] Leon AC, Davis LL, Kraemer HC (2011). The role and interpretation of pilot studies in clinical research. J Psychiatr Res.

[22] Tandon R, Yasmin N, Baxla M, Krishna H (2025). Developing concept maps as an aid for enhancing analytical thinking in undergraduate medical students. Cureus.

